# Azurin/CdSe-ZnS-Based Bio-Nano Hybrid Structure for Nanoscale Resistive Memory Device

**DOI:** 10.3390/ma10070803

**Published:** 2017-07-15

**Authors:** Ajay Kumar Yagati, Taek Lee, Jeong-Woo Choi

**Affiliations:** 1 School of Integrative Engineering, Chung-Ang University, Heukseok-dong, Dongjak-gu, Seoul 06974, Korea; ajay.yagati@gmail.com; 2 Department of Chemical & Biomolecular Engineering, Sogang University, Shinsu-dong, Mapo-gu, Seoul 04107, Korea; nanotlee@gmail.com; 3 Department of Chemical Engineering, Kwangwoon University, 20 Gwangun-Ro, Nowon-Gu, Seoul 01897, Korea

**Keywords:** azurin, CdSe-ZnS, scanning tunneling spectroscopy, bio-nano hybrid structure, resistive bistable switching

## Abstract

In the present study, we propose a method for bio-nano hybrid formation by coupling a redox metalloprotein, Azurin, with CdSe-ZnS quantum dot for the development of a nanoscale resistive memory device. The covalent interaction between the two nanomaterials enables a strong and effective binding to form an azurin/CdSe-ZnS hybrid, and also enabled better controllability to couple with electrodes to examine the memory function properties. Morphological and optical properties were performed to confirm both hybrid formations and also their individual components. Current-Voltage (*I–V*) measurements on the hybrid nanostructures exhibited bistable current levels towards the memory function device, that and those characteristics were unnoticeable on individual nanomaterials. The hybrids showed good retention characteristics with high stability and durability, which is a promising feature for future nanoscale memory devices.

## 1. Introduction

Azurin, which is a blue copper redox protein, plays a key role in transporting the electrons in many important biological reactions, such as photosynthesis, and in bacterial respiratory redox-chains [1,2]. The electron transfer process occurs through the copper-containing active site, which is characterized by an intense ligand-to-metal charge transfer absorption band located around 628 nm [3]. Further, azurin is one of the most widely studied proteins, due to its excellent electrochemical properties and its ability to form conjugations with various proteins and nanoparticles [4,5]. Additionally, the utilization of metallic and semiconductor nanoparticles (NPs) has gained much importance in various fields such as electrocatalysis, and as a biological marker in disease diagnostics [6]. Due to size-dependant electronic properties [7], NPs have gained much technological importance, making the material important in many aspects of study in molecular electronics [8] and biomedical engineering [9]. Recently, biomolecules coupled with nanoparticles have been studied in detail in order to modulate their conductive properties [10], and also for the enhancement of local electromagnetic fields [11].

Among various nanomaterials, quantum dots (QDs) have gained much importance because of their numerous applications in optoelectronics [12,13], sensors [14], drug delivery [9,15,16] and memory devices [17]. Specifically, core-shell QDs, with diameters ranging from one nanometer to tens of nanometers and an outer ‘shell’ of capping ligands, have been chemically modified to carry a host of functional groups for attachment to biological molecules. The conjugation of biomolecules with CdSe-ZnS nanoparticles has been achieved by covalent bond formation via different linker molecules [18] and with the help of electrostatic interaction in self-assembled cases [19]. Recently, CdSe-ZnS quantum dots coupled with bio/organic molecules based structures have shown electrical bistability with good charge retention characteristics, during current-voltage (*I-V*) sweep measurements for future memory applications [20]. Generally, in semiconductor nanoparticles, electrical bistability arises due to the charge confinement [21]; however, in bio-nano hybrid molecules, the conformational change or the electro-reduction of molecules plays a key role in changing the conductivity of the molecules [22], which has led to the development of advanced electronic devices with repeatable conductance switching applications. Thus, formation of bio-hybrid nanostructures by fine-tuning the surface properties results in excellent electronic and optical properties for an enormous range of applications such as sensing, imaging, delivery, catalysis and memory devices. Hence, hybrid structures containing inorganic nanoparticles are currently emerging as excellent candidates for potential applications in next-generation nonvolatile memory devices [20,23].

The present work focuses on the creation of a new type of bio-nano hybrid substrate for nanoscale memory applications. Specifically, it is shown that the nanoscale interactions of metalloprotein and colloidal semiconductor quantum dots can be utilized as a nanoscale memory device. To test the electrical response of the hybrid system and to assess their applications in bionanodeivces, we combined blue copper protein azurin and CdSe-ZnS quantum dots on an Au electrode. The use of azurin in direct communication with the electrode provides the basis for electron transport for memory applications [24,25]. Recently, we proposed a nanoscale resistive random access memory device based on recombinant azurin and CdSe-ZnS particles [20]; however, there are drawbacks to making this structure, such as the long time and complicated steps required to generate the hybrid structures using recombinant DNA technology. Furthermore, complex design skills were required to produce recombinant engineered protein at high yield in order to develop these bio-nano hybrids. Thus, a simple, robust and easy way to produce bio-nanostructures with high durability and endurance is required.

**Figure 1:**
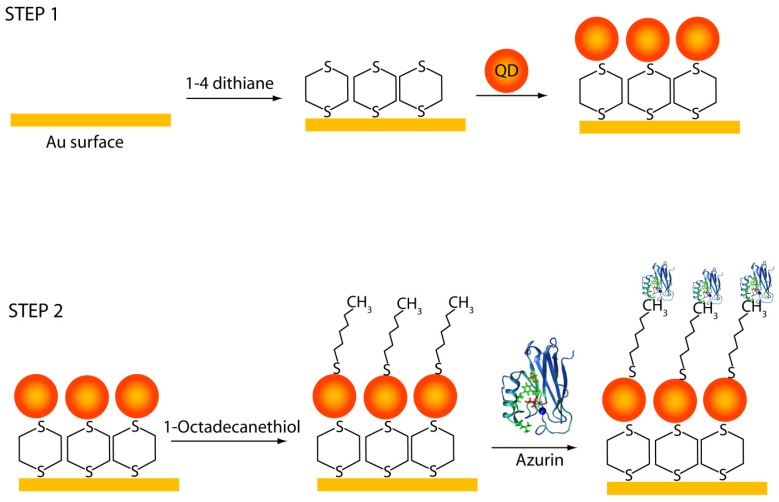
(**Step 1**) Immobilization scheme of CdSe-ZnS on an Au electrode via the ligand 1,4 Dithiane; and (**Step 2**) Immobilization strategy for azurin with CdSe-ZnS on an Au electrode where azurin was adsorbed onto a 1-octadecanethiol self-assembled monolayer, thereby forming a single bio-nano hybrid nanostructure.

Therefore, in this work we develop a robust and simple way to fabricate bio-nano hybrids for memory function, and thus we follow the step-by-step procedure for the conjugation of azurin with CdSe-ZnS quantum dots shown in Figure 1. The azurin/CdSe-ZnS hybrid was characterized using surface plasmon resonance (SPR), scanning tunneling microscopy (STM) and transmission electron microscopy (TEM). The charge transfer effect in the azurin/CdSe-ZnS nanoparticle hybrid, as well as the charge transfer induced conductance switching required for operation of the electronic memory devices, were explained. Current–voltage (*I–V*) measurements were carried out to investigate the electrical properties of azurin and CdSe-ZnS individually, as well as of the azurin/CdSe-ZnS hybrids, on the Au electrode. The stability of the device was also confirmed with repeatable measurements of *I–V* characteristics from the hybrid structures.

## 2. Results and Discussion

### 2.1. Optical Characterization of Azurin and CdSe-ZnS Hybrids

**Figure 2:**
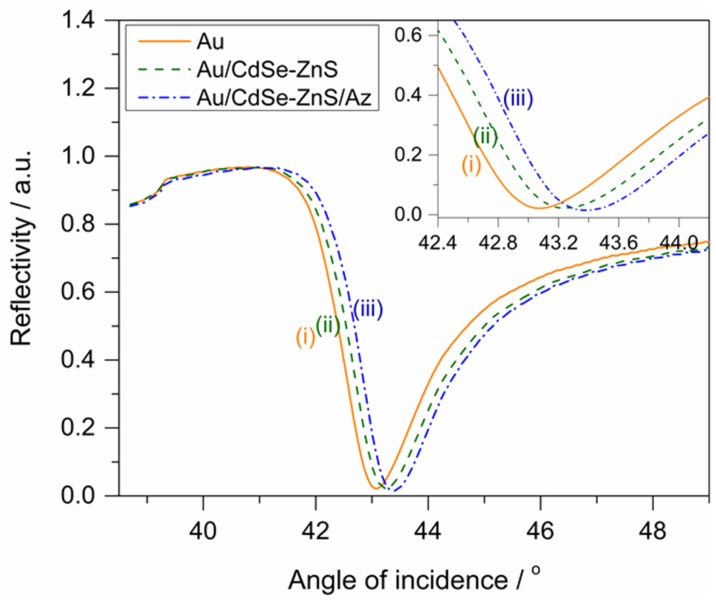
Sets of experimental SPR curves averaged over several locations on an electrode corresponding to (**i**) bare Au; (**ii**) CdSe-ZnS on Au; and (**iii**) Azurin/CdSe-ZnS/Au. Inset shows the zoomed portion of the reflective angle from 42.4° to 44.4°.

To confirm the binding of azurin with CdSe-ZnS quantum dots, surface plasmon resonance (SPR) spectroscopy was used for the binding process of each molecular interaction formed on the Au electrode. The binding process of the azurin with CdSe-ZnS on the electrode Au was confirmed via the shift in the reflectivity angle with respect to the electrode. Several SPR curves were measured at different locations of the bare Au, the CdSe-ZnS bound electrode and the azurin coupled with CdSe-ZnS electrode, which averaged as shown in Figure 2. The spectrum clearly shows an increase in reflective angle position for each modification on the Au electrode. The inset of Figure 2 shows a zoomed portion, from which we can clearly observe that the bare Au electrode possessed a reflective angle of 43°, and a shift of 0.2° in the minimum reflectivity angle of the spectrum was observed for CdSe-ZnS immobilied Au electrode, and 0.4° shift in reflective angle was observed in the case of azurin/CdSe-ZnS hybrid molecules immobilized Au electrode. This suggests that the azurin/CdSe-ZnS hybrid was well formed on the electrode. Thus, SPR angle measurements provide good support for the successful surface characterization of hybrids on an Au electrode.

**Figure 3:**
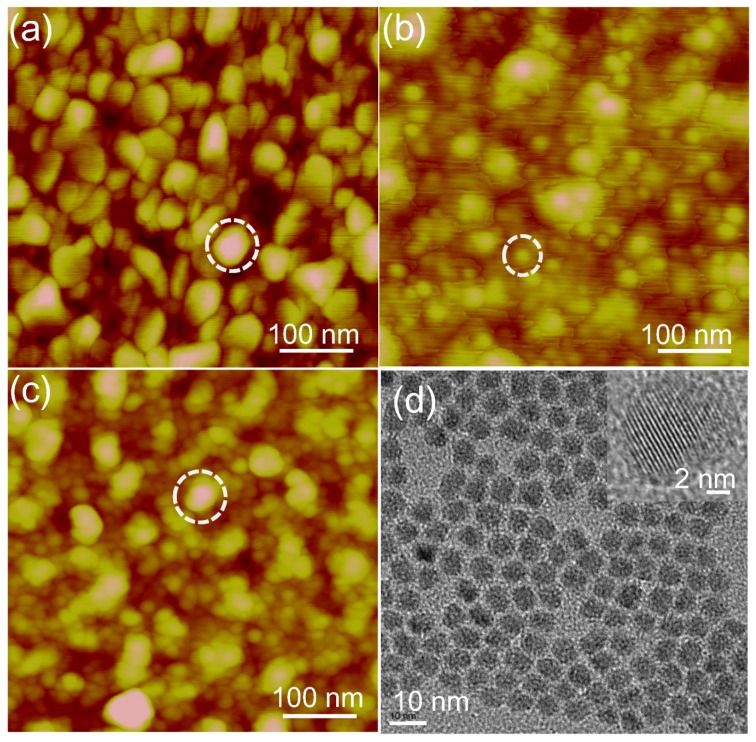
The morphology images obtained from scanning tunneling microscopy for (**a**) bare Au; (**b**) CdSe-ZnS on Au; and (**c**) azurin/CdSe-ZnS/Au. Images were obtained at a scan rate of 1 Hz. The section analysis shows the corresponding height and width of the particles of each obtained image. (**d**) HR-TEM image of CdSe-ZnS nanoparticles inset shows the lattice structure of a single CdSe-ZnS particle.

Scanning tunneling microscopy (STM) was used to characterize the morphology of azurin/CdSe-ZnS hybrids on an Au electrode. The images clearly show that the electrodes represent different morphologies with characteristic surface textures. The bare Au electrode was found to have rough ridges, as shown in Figure 3a. The image was scanned at a rate of 1 Hz and circled area indicated in the images was considered for section analysis (not shown here). From the section analysis, it was found that the bare Au consists of ridges with a length of 43 nm. The CdSe-ZnS particles shown in Figure 3b measured to be ~20 nm in size but, the size of the QD particle looks bigger which may be due to the tip convolution effects otherwise a well distibuted QD surface was obtained by covalent linking procedure. The morphology of the azurin linked CdSe-ZnS hybrid system is shown in Figure 3c, which clearly shows both of the nanomaterials integrating as a hybrid. These hybrids were measured to be 42 nm, which means that the protein molecules were well attached to the CdSe-ZnS, thereby creating a hybrid molecular structure. However, there is a chance for direct linkage of the azurin protein to the Au electrode by 1-octadecanethiol; however, the conjugated part of azurin/CdSe-ZnS was selected for the present study. TEM images of the core-shell nanoparticles are presented in Figure 3d, which shows isolated particles over the entire electrode; High-resolution transmission electron microscopy (HRTEM) image of the core-shell nanoparticle shows a lattice spacing of the CdSe core of 0.22 nm. The size of the core-shell nanoparticles, as estimated from HR-TEM images over a large area, was estimated to be about 5 nm.

### 2.2. Electrical Properties of Azurin, CdSe-ZnS and Azurin/CdSe-ZnS on an Au Electrode

**Figure 4:**
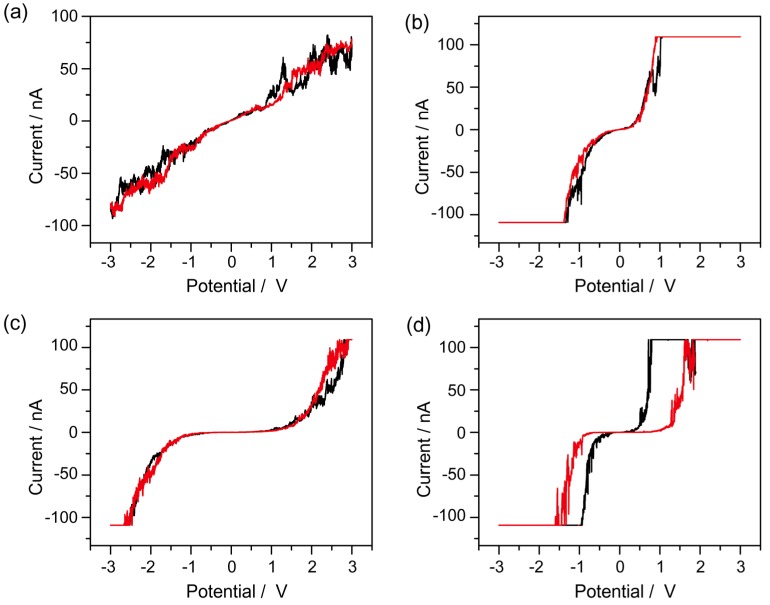
*I–V* characteristics obtained from scanning tunneling spectroscopy (STS) for (**a**) bare Au; (**b**) CdSe-ZnS on Au; (**c**) azurin on Au; and (**d**) azurin/CdSe-ZnS on an Au electrode respectively.

Scanning tunneling spectroscopy (STS) curves on single bio-nano hybrid were measured under an open-air environment in ambient conditions by directly positioning the STM tip (Pt/Ir) on a single azurin/CdSe-ZnS hybrid, azurin/Au, CdSe-ZnS/Au and on bare Au, respectively. All measurements were carried out by first taking a morphology scan of a monolayer of assembled azurin/CdSe-ZnS hybrid, and then driving the STM tip to the top of a single hybrid, where a measurement was directly taken across the azurin/CdSe-ZnS. To examine the properties at nanoscale, an Au/Si substrate was used as the bottom electrode, while the 14 mm length of the conductive STM tip was used as the top electrode, with a single azurin/CdSe-ZnS hybrid sandwiched in between both contacts. *I–V* curves were measured as the tip-sample bias was ramped in a range of ±3 V. The shape of the *I–V* curve depends on the STM tip position over the azurin molecule where the current is measured. The bare Au didn’t show any peculiar behavior, and showed a linear characteristic governed by ohms law as shown in Figure 4a. However, in the case of CdSe-ZnS/Au and azurin/Au, the spectra were found to be nonlinear [26], as shown in Figure 4b,c, forming a double-tunnel junction configuration, such as STM tip/azurin or CdSe-ZnS/conducting Au substrate. Although there are slight fluctuations in the *I–V* curves of CdSe-ZnS and azurin, which depend on many factors, such as whether or not the molecule is a donor-acceptor pair, schottky barrier effects, and on the air gap between the tip and molecule. Considering no apparent coulomb blockade with nanoparticles of size 15 nm [27], the CdSe-ZnS/Au and azurin/Au systems revealed a rectifying-like behavior of the metal/molecule/metal tunneling junction, and no memory effects were observed from the individual components.

In order to demonstrate that the observed electrical bistability of the azurin/CdSe-ZnS hybrid is due to the conjugation of azurin and CdSe-ZnS, rather than an electronic phenomenon caused by either one of the two nanomaterials composing the hybrid, we conducted electrical studies on azurin and CdSe/ZnS as controls. As Figure 4b,c shows, that conductance switching is absent in both control samples, supporting the conclusion that the conjugation between both nanomaterials (semiconducting nanoparticles and insulating linker with azurin) is important for the observed memory effect.

**Figure 5:**
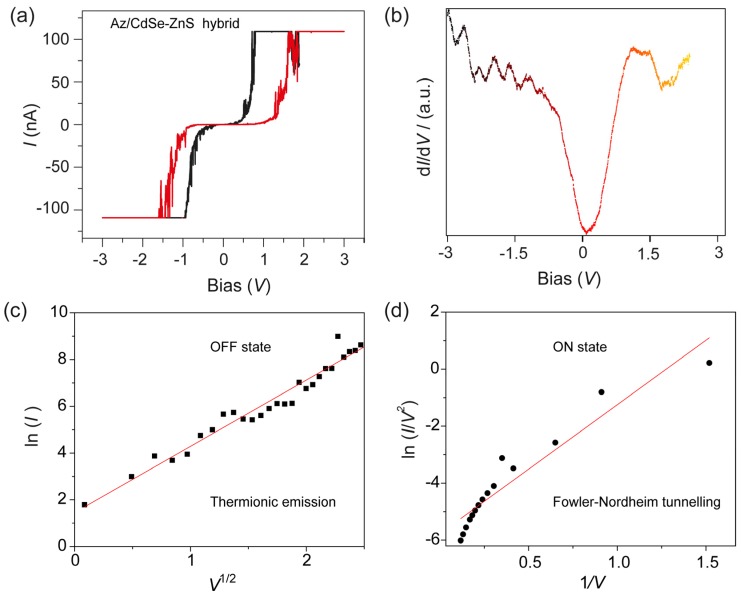
(**a**) *I–V* characteristics of the azurin/CdSe-ZnS hybrid on Au, shows low (OFF state) to high (ON state) for memory device applications; (**b**) d*I*/d*V* vs *V* spectra on the azurin/CdSe-ZnS on the Au electrode; (**c**) Plot of ln(*I*)–*V*^1/2^ fit for the low conducting state, and; (**d**) ln(*I*/*V*^2^)–1/*V* fit for the high conducting state.

*I–V* characteristics of a monolayer of azurin/CdSe-ZnS on Au measured with a Pt/Ir tip of STM under two sweep directions are shown in Figure 5a. A memory effect can be observed when the trace and retrace linear voltage scans are applied from −3.0 to +3.0 V respectively. As observed, the azurin/CdSe-ZnS hybrid is initially in a low conductivity state (defined as the OFF state), until it reaches about −1.5 V, where an abrupt increase in conductivity occurs (defined as the ON state), indicating a transition of the device from an initial OFF state to an ON state, equivalent to the “writing” process in a digital memory device. It remains in a high conductive state as the voltage increases above 1.5 V. Thus the threshold voltage (V_Th_) for switching is 1.5 V. When a negative voltage is applied, the device is returned to a low conductivity state (OFF state) with reverse voltage bias; equivalent to the “erase” state of the device. The different conduction mechanisms in the two states suggest a change in the electron distribution of the device after the electrical transition. The transition from low to high conductivity states took place in nanoseconds, and we expect that the redox protein behaves as the charge donor during the conductivity switching, allowing charge transfer to the lower-energy core. Hence, there is an electric field-induced charge transfer mechanism between azurin and the CdSe–ZnS core for the observed memory phenomena. The first derivative *I-V* measurements on azurin/CdSe-ZnS electrode was shown in Figure 5b, which reaveals that the hybrid structure has the bandgap around 1 eV.

### 2.3. Experimental Fit for ON-OFF Currents of Azurin/CdSe-ZnS Hybrid Layer on an Au Electrode

To study the transport or conduction mechanisms involved in the ON and OFF states, we fitted the *I–V* characteristics with theoretical models. The current in the OFF state is basically due to therminonic emission associated with thermally activated charge injection from the electrode to the azurin/CdSe-ZnS. The current in the OFF state shows a linear relation for ln(*I*) vs. *V*^1/2^. (Figure 5c). This OFF-state current was mostly considered to be leakage current before the switching transistion occurs. Additionally, the conduction current in the ON state was fitted using the Flower-Nordheim tunnelling model. At higher voltages (>0.3 V), a linear relation of ln(*I*/*V*^2^) vs. 1/*V* was observed (Figure 5d). Therefore, in the ON state, the conduction mechanism is no longer thermionic injection as in the OFF state, but charge tunnelling.

Thus, the sudden transistion of the current in the *I–V* measurements from the azurin/CdSe-Zns system is due to the charge transfer from the azurin protein to the CdSe-ZnS under the influence of the high electric field, which changes the conductivity of the hybrid system. Once the electrons are transferred from azurin (azurin contains a copper ion which is able to switch between two states (Cu^I^/Cu^II^)), it will be trapped in the CdSe core and stabilized by the outer ZnS shell. As this is an induced electrical field effect, it was observed that a sufficient number of sites were established to enable a sudden jump of current accompanying the charge tunnelling through the nanoparticles. The activation energy of charge trapping and charge retention are strong evidence for the trapping behavior of CdSe-ZnS nanoparticles. After the charge was de-trapped from the nanoparticles and returned to the azurin, the conductivity of the hybrid dropped to the OFF state. These interactions between the azurin and CdSe-ZnS core-shell augment the *write* and *erase* processes in the memory device, while this kind of switching behavior was not observed in azurin and CdSe-ZnSonly devices. The hybrid structure therefore plays a role as charge donor and in stabilizing the charges, creating a repeatable memory effect in azurin/CdSe-ZnS devices.

### 2.4. Robustness of the azurin/CdSe-ZnS Hybrid Device

The device ON and OFF switching cycles on the azurin/CdSe-ZnS-based memory device were performed by applying a *write* pulse of 1.5 V and an *erase* pulse of 0.5 V, both with a time width of 10^−^^3^ s, and which were used to turn the device ON and OFF, respectively. The device can be programmed for 50 cycles with an ON/OFF ratio of around 1000. Thus, the developed device shows high stability and shows a repeatable hysteresis curve, and the performance of the device is better than the recombinant azurin/CdSe-ZnS device [20], which required a voltage greater than 2.0 V to switch the device ON. Furthermore, the performance of the device can be easily fine-tuned by incorporating various types of CdSe-ZnS nanoparticles that demonstrate a size-dependent carrier relaxation process [7]. Hence, the charge retention capability and endurance of the device can be greatly improved by tuning the size of the nanoparticles, and also by utilizing various types of metalloprotein instead of azurin. These alteration will show a change in threshold voltages and also chemical linkers with different chain lengths can fine tune the dipole interactions between the nanoparticles and the azurin protein.

## 3. Materials and Methods

### 3.1. Materials 

For STM measurements, gold (Au) substrate was used as the bottom electrode, which was purchased from Inostek (Gyeonggi-do, Korea). The fabrication of the Au substrate included a *p*-type silicon substrate which was used as a solid support. A SiO_2_ layer that was 3000 Å thick was grown by thermal oxidation. A chromium (Cr) layer that was 20 Å thick was then sputtered onto substrate as an adhesion material, which was followed by Au (111) sputtering to a thickness of 430 Å. For SPR measurements, Au substrate sputtered on a 2 nm Cr deposited cover glass BK7 (Inostek, Gyeonggi-do, Korea) was utilized.

The CdSe-ZnS nanoparticles were purchased from Lumidot™, Sigma Aldrich (St. Louis, MO, USA). Pseudomonas aeruginosa azurin (MW 14.0 kDa) was purchased from Sigma Aldrich and was used without any further purification. 1, 4-Dithiane (97%), mercaptosuccinic acid (97%) and 3-mercaptopropionic acid (≥99%) were purchased from Sigma Aldrich. Ethanol (70%), H_2_SO_4_ and 30% H_2_O_2_ were purchased from Daejung Chemicals and Metals Co. Ltd. (Gyeonggi-do, Korea). Buffer solutions were prepared using ultrapure deionized (DI) water (18.2 MΩ/cm) supplied by a Milli-Q system (Millipore, Bedford, MA, USA). Deionized water was used for cleaning the substrates and high-purity N_2_ gas was used for drying those cleaned substrates. All other reagents were of analytical reagent grade, or of the highest purity available, and used without further purification unless indicated otherwise.

### 3.2. Formations of Azurin/CdSe-ZnS/Au Hybrid Structures

Au substrates were kept in piranha solution (1:3) v/v, H_2_O_2_ and concentrated H_2_SO_4_ for 4 min and rinsed thoroughly with distilled water. After drying with nitrogen gas, the cleaned substrates were immersed in saturated dithiane solution in ethanol at 65 °C for 3 days with an intense ethanol rinsing afterwards. For immobilizing CdSe-ZnS, the cleaned Au substrates were immersed in a CdSe-ZnS mixed chloroform suspension for 2 days at 50 °C. Azurin was then immobilized by using the procedures described in the literature [28,29]. The CdSe-ZnS-modified Au electrode was incubated in 5 mM octadecanethiol (ethanol solution) and left to react for 24 h at 50 °C. After washing with ethanol and DI water, the modified QD on the Au electrode was immobilized with 0.5 mg/mL azurin in 10 mM HEPES buffer (pH 7.0) for 3 h. The coupling with octadecanethiol provided the copper redox center of the azurin faces to the ZnS outer shell in hydrophobic adsorption mode [30].

### 3.3. Electrode Characterization of Azurin/CdSe-ZnS/Au Hybrids

To confirm the immobilization of the azurin/CdSe-ZnS and CdSe-ZnS on the Au electrode, surface plasmon resonance (SPR) was used where the intensity of the reflected light is measured as a function of the angle of incidence. A 150-W quartz tungsten–halogen lamp (Schott, Mainz, Germany) was used as the light source, and the light was delivered to a goniometer arm (Physik Instrumente, Karlsruhe, Germany). The angle position of this minimum depends on the thickness and optical properties of the layer on the gold substrate. The STM measurements were conducted by using Nanoscope (R) III (Digital instruments, Santa Barbara, CA, USA). Platinum tips of 14 mm were purchased from Veeco Korea instruments (Seongnam, Gyeonggi-do, Korea), and images were obtained with a set point current and bias voltage of 0.5 nA and 100 mV respectively. The morphology and crystal structure of CdSe-ZnS nanoparticles were investigated by high resolution transmission electron microscopy (HRTEM) using a JOEL JSM instrument at an operating voltage of 200 kV. TEM samples were prepared by depositing a few drops of the CdSe-ZnS solution on a carbon-coated copper grid. Current–voltage (*I–V*) spectra were recorded by positioning the tip on top of the redox protein and protein-nanoparticle hybrid, and then the feedback loop was disengaged. The tunneling current was monitored by sweeping the bias within a range of ±3.0 V.

## 4. Conclusions

The present study demonstrated an easy and robust method for hybrid formation and characterized the electrical resistive properties along with memory effects observed in azurin/CdSe-ZnS bio-nano hybrid on an Au electrode. The effective binding of azurin, CdSe-ZnS and azurin/CdSe-ZnS on Au electrodes was achieved by covalent linking chemistry for attachment on an Au electrode. The formation of the hybrids was characterized SPR, STM, and TEM. Experiments on the electrical properties of the bio-nano hybrid of azurin/CdSe-ZnS exhibited excellent electrical bistability and memory phenomena. The conductive properties of the individual molecules measured in air showed a rectifying-like behavior with no memory effect. The ON/OFF ratio of the hybrid device was in the range of 10^3^. The developed device shows better performance in terms of device switching (V_Th_ < 1.5 V) and charge retention, thereby showing repetitive hysteresis curves comparative to recombinant azurin/CdSe-ZnS structures. Electrically bistable resistive memory devices fabricated utilizing azurin/CdSe-ZnS hybrid hold promise for potential applications in next-generation non-volatile memory devices, which is an interesting field of study in view of developing future nanoscale biomemory devices.
